# UCP2 overexpression enhanced glycolysis via activation of PFKFB2 during skin cell transformation

**DOI:** 10.18632/oncotarget.20762

**Published:** 2017-09-08

**Authors:** Annapoorna Sreedhar, Petra Petruska, Sumitra Miriyala, Manikandan Panchatcharam, Yunfeng Zhao

**Affiliations:** ^1^ Department of Pharmacology, Toxicology & Neuroscience, LSU Health Sciences Center in Shreveport, Shreveport, LA 71130, USA; ^2^ Department of Anatomy and Cell Biology, LSU Health Sciences Center in Shreveport, Shreveport, LA 71130, USA

**Keywords:** mitochondrial uncoupling 2, PFKFB2, mitochondria, glycolysis, skin cells

## Abstract

Uncoupling protein 2 (UCP2) is an inner mitochondrial membrane transporter which is often upregulated in human cancers. However, how this anion transporter affects tumorigenesis is not well understood. Using the skin cell transformation JB6 model, we demonstrated that UCP2 overexpression activated phosphofructokinase 2/fructose-2,6-bisphosphatase 2 (PFKFB2), a key regulator of glycolysis. In conjunction, upregulation of PFKFB2 expression correlated with elevated fructose 2,6-bisphosphate (Fru-2,6-P_2_) levels, 6-phosphofructo-1-kinase (PFK-1) activity, glucose uptake, and lactate production. Inhibiting PFKFB2 expression suppressed UCP2-mediated skin cell transformation, decreased cell proliferation, and enhanced mitochondrial respiration, while dampening aerobic glycolysis. The AKT signaling pathway was activated in the UCP2 overexpressed cells; furthermore, the activated AKT signaling contributed to the activation of PFKFB2. Whereas AKT inactivation blocked PFKFB2 activation, suggesting that AKT activation is an important step in PFKFB2 activation. Collectively, our data suggest that UCP2 is a critical regulator of cellular metabolism during cell transformation. Our data also demonstrate a potentially novel mechanism to understand UCP2's tumor-promoting role, which is through the AKT-dependent activation of PFKFB2 and thereby, the metabolic shift to glycolysis (the Warburg effect).

## INTRODUCTION

Unlike normal cells, cancer cells are characterized by uncontrolled growth, enhanced proliferation, and replicative immortality [1]. Cancer cells often reprogram their metabolism to fuel their accelerated growth [2]. This observation was first made by Otto Warburg, one of the pioneers in the field of cancer metabolism, who claimed that the root cause of cancer is too much acidity in the body [3–5]. In other words, the Warburg effect is the observation that cancer cells heavily depend on glycolysis leading to lactic acid production even in the presence of oxygen [6–7]. However, the definitive reason for this observation remained debatable [8–9].

According to a recent review by Hanahan and Weinberg [1], much attention has been given to a dysregulated metabolism as an emerging hallmark of cancer cells with a primary focus directed at the dependence of tumor cells on enhanced glycolysis [10–13]. This realization has brought renewed attention to the Warburg effect, and this metabolic alteration of tumor cells is being extensively studied.

Cancer cells undergo various metabolic and autonomous changes that drive tumorigenesis [11, 14]. In addition to our work, other studies have also suggested that one such driver of tumorigenesis is the mitochondrial uncoupling protein 2 (UCP2) [15–16]. Uncoupling proteins are a family of anion transporters present in the inner mitochondrial membrane whose protein expressions are closely related to changes during cell proliferation and tumorigenesis [16–19]. We and others have demonstrated that UCP2 is often highly expressed in human cancers [20–22]. Although the exact role of UCP2 during carcinogenesis remains elusive, studies from our lab (manuscript under review) and others’ research demonstrate that UCP2 is a crucial player in the cascade of mitochondrial molecular events, redox regulation, cell cycle, cell proliferation, cell survival, and apoptosis [23–26]. Since UCP2 overexpression is closely associated with enhanced cell proliferation and tumorigenesis, targeting UCP2 expression may be a winning therapeutic strategy to treat UCP2 overexpressed cancers. Moreover, it was recently reported that UCP2 is not just an uncoupling protein but also a metabolite transporter [27]. This observation would put UCP2 in a position to influence cellular metabolism in favor of tumorigenesis. Hence, it is important to understand the molecular basis of UCP2 overexpression in tumor cell metabolism, differentiation, and survival. Therefore, in an attempt to understand how UCP2 regulates cellular energy metabolism during skin carcinogenesis, we performed a protein microarray analysis using the skin tumor tissues obtained from wild-type and UCP2 knockout mice at the end of a skin carcinogenesis study [28]. In this study, we identified that PFKFB2 was upregulated in skin tumor tissues of the wild-type mice but not in the UCP2 knockout mice, suggesting that PFKFB2 expression may be positively regulated by UCP2 [28]. PFKFBs are a family of bifunctional enzymes that control the levels of fructose 2,6-bisphosphate (Fru-2,6-P_2_). They are termed ‘bifunctional’ because they catalyze the synthesis and the degradation of Fru-2,6-P_2._ Fru-2,6-P_2_ is a powerful allosteric activator of 6-phosphofructo-1-kinase (PFK-1), a key enzyme in controlling the glycolytic flux. PFK-1 catalyzes the conversion of Fru-6-P (fructose 6- phosphate) into Fru-1,6-P_2_. [29–30]. Therefore, increased levels of Fru-2,6-P_2_ would allow cells to maintain a high glycolytic flux. In mammals, there are four isoforms of PFKFB genes (PFKFB1, PFKB2, PFKFB3, and PFKB4) which are characterized by tissue expression [31]. In fact, PFKFB2, PFKFB3, and PFKFB4 are overexpressed in the majority cancers, leading to a higher concentration of Fru-2,6-P_2_ and increased glycolytic flux to lactate [32–34].

Given that PFKFB2 was activated in wild-type mice but not in UCP2 knockout mice during the skin carcinogenesis study [28], we speculated that UCP2 overexpression may bring a metabolic shift towards glycolysis by activating PFKFB2 as a potential novel mechanism of tumor promotion. Utilizing the well-established JB6 skin cell transformation model, we have performed mechanistic studies to determine whether PFKFB2 activation is important for skin tumorigenesis and how UCP2 overexpression regulated PFKFB2 activation.

## RESULTS

### Glycolysis versus mitochondrial respiration in UCP2 overexpressed cells

**Figure 1:**
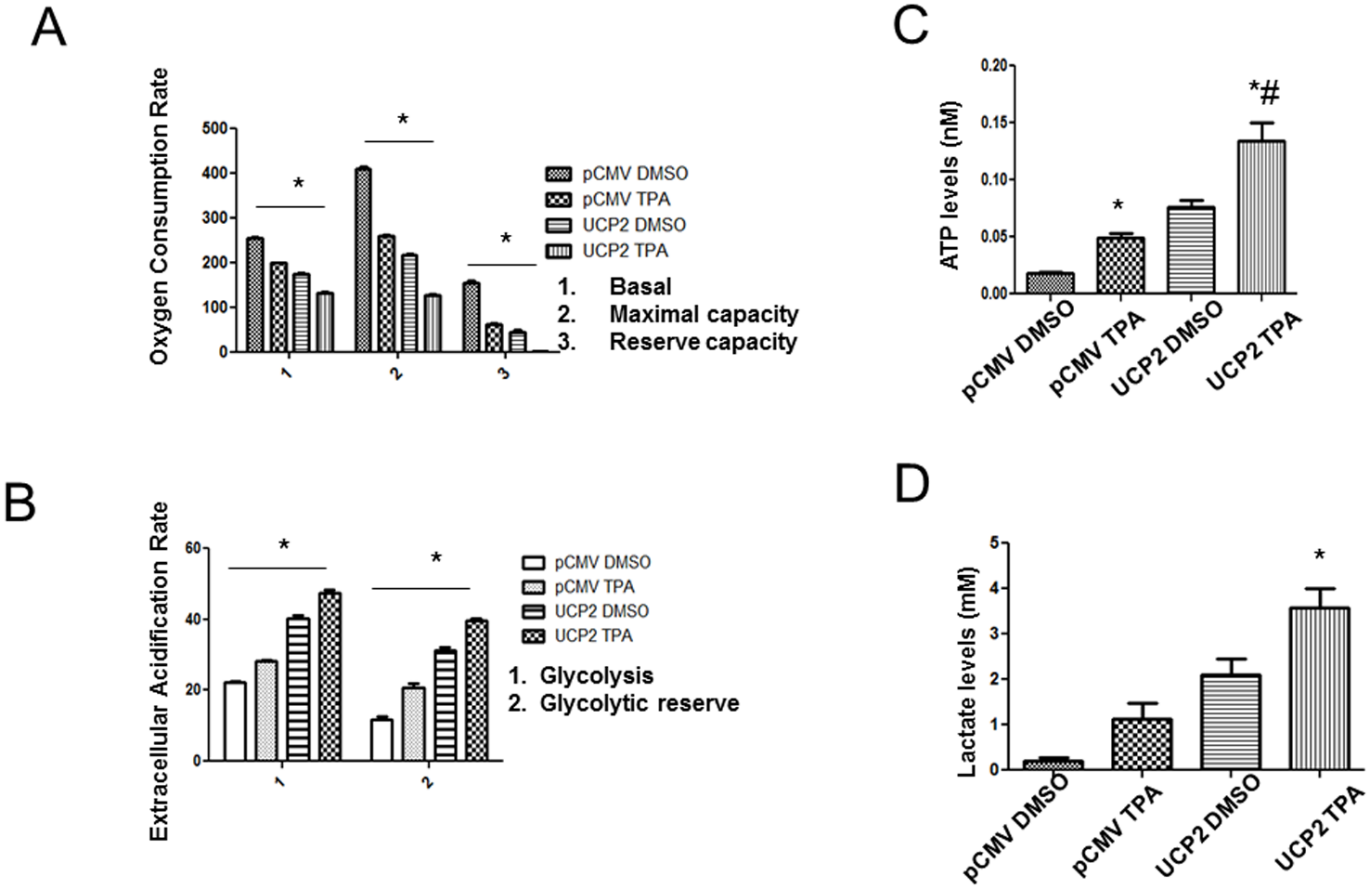
UCP2 overexpression promotes glycolytic metabolism (i) Cellular bioenergetics was measured using the Seahorse Bioscience XF Analyzer. 50,000 cells/well were seeded in the Seahorse plate. **(A)** Oxygen consumption rate (OCR), an indicator of mitochondrial oxidative phosphorylation and **(B)** extracellular acidification rate (ECAR), an indicator of glycolysis, were measured using control pCMV cells and UCP2 overexpressed cells. UCP2 overexpressed cells had decreased OCR and an increased ECAR compared with control pCMV cells, suggesting that UCP2 overexpression promotes cellular adaptation towards enhanced glycolysis. Results were an average of three independent experiments. Statistical analysis was performed using two-way ANOVA with test. ^*^p<0.001 (N=3). (ii) Furthermore, glycolytic rates were assessed by measuring cellular ATP levels **(C)** and lactate levels **(D)** in control pCMV and UCP2 overexpressed cells. Filtered whole cell lysates were used in the experiments. Cellular ATP and lactate levels were normalized to protein concentration. Lactate levels and ATP levels were significantly higher in the UCP2 overexpressed cells compared to the control pCMV cells. n=4 per group, data are presented as the mean ± SD. ^*^, p<0.05 when compared with its own control/DMSO group; ^#^, p<0.05 when compared with the control/TPA group.

To understand how upregulated UCP2 affects cellular bioenergetics, oxygen consumption rate (OCR) and extracellular acidification rate (ECAR), as indicators of mitochondrial respiration and glycolytic rate, respectively, were measured. The measurements were taken using a Seahorse Bioscience XF Analyzer in control pCMV cells and UCP2 overexpressed cells [35–36]. Modulators of oxidative phosphorylation such as oligomycin, FCCP, and antimycin/rotenone were added sequentially to the cells and OCR was determined. The basal OCR rates were 255 pmol/min in pCMV cells and 170 pmol/min in UCP2 overexpressed cells; and TPA treatment further reduced the basal OCR rates (Figure 1A). To determine the maximal capacity of these cells, p-trifluoromethoxy carbonyl cyanide phenyl hydrazone (FCCP), an uncoupler was added to the respiring cells. The maximal respiratory rate as determined by FCCP was significantly lower in the UCP2 overexpressed cells compared to the control pCMV cells (Figure 1A). Mitochondrial reserve capacity, the amount of oxygen consumption that is available for cells to use in times of stress or increased ATP demand, was calculated by subtracting basal respiration from maximal respiration (FCCP). As shown in Figure 1A, reserve capacity was significantly decreased in the UCP2 overexpressed cells compared to the control pCMV cells and TPA treatment further reduced the reserve capacity. These results show that OCR is significantly dampened in UCP2 overexpressed cells. To determine whether the decreased OCR was compensated with an elevated glycolytic rate, ECAR was also measured in these cells. As shown in Figure 1B, in contrast to the OCR results, glycolysis was at maximum in the presence of glucose in the UCP2 overexpressed cells and TPA treatment further elevated the glycolytic rates. Basal glycolysis, glycolytic capacity, and glycolytic reserve in UCP2 overexpressed cells were significantly higher than that of control pCMV cells. Therefore, both the OCR and ECAR data together suggest that mitochondrial respiration is dampened, whereas glycolysis is enhanced in UCP2 overexpressed cells; therefore, UCP2 overexpression may cause a switch from mitochondrial respiration to glycolysis. Furthermore, intracellular ATP levels were elevated in the UCP2 overexpressed cells (Figure 1C), suggesting that UCP2 overexpression may serve as an important bioenergetic regulator which enhances glycolysis to produce more ATP to meet the need for proliferation. The increase in the levels of lactate (Figure 1D) in the UCP2 overexpressed cells is further confirmation of the Warburg effect observed in UCP2 overexpressed cells.

### PFKFB2 is activated in UCP2 overexpressed cells

**Figure 2:**
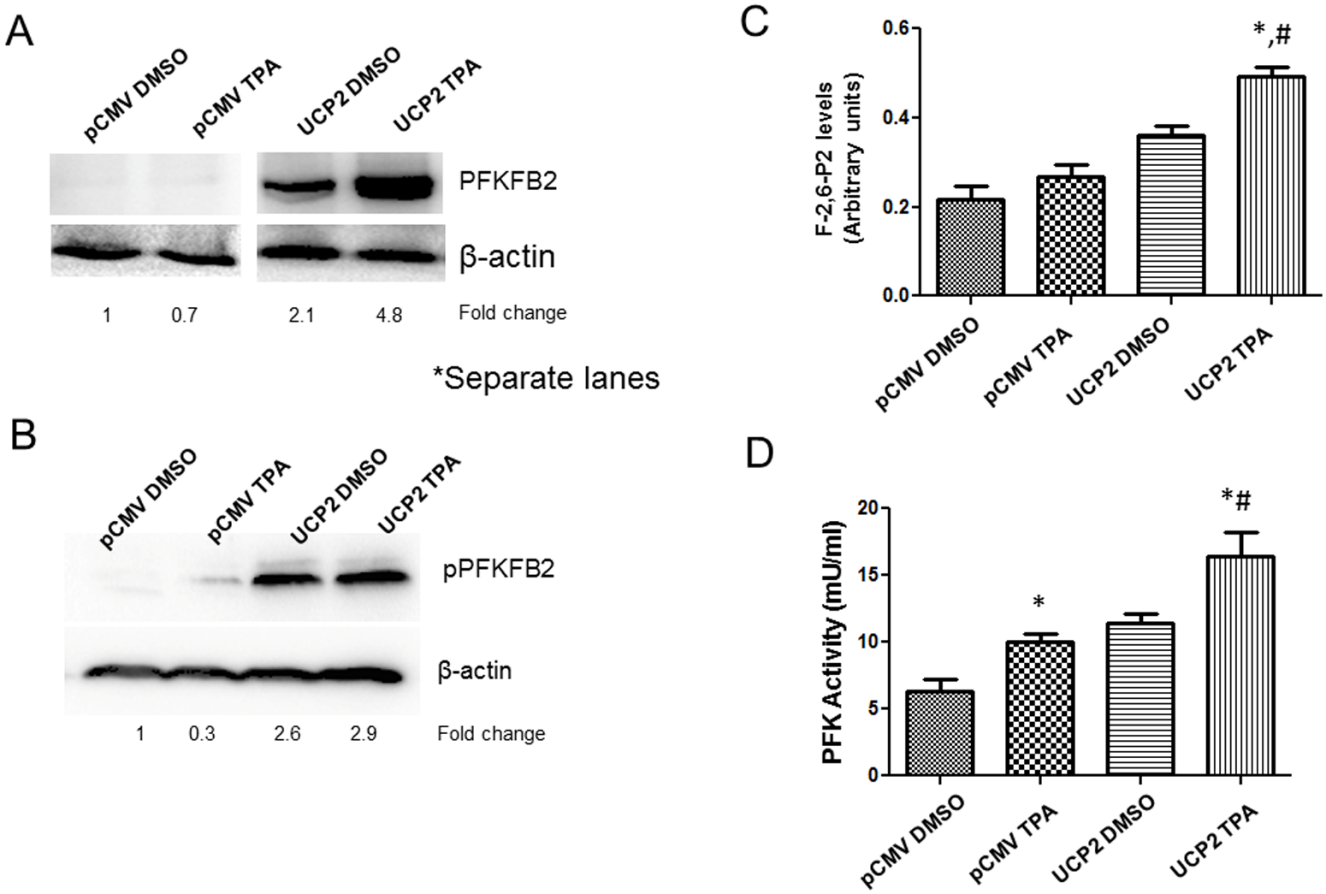
Upregulation of PFKFB2 expression in UCP2 overexpressed cells (i) Western blot analysis of PFKFB2 **(A)** and pPFKFB2 **(B)** expression levels in control pCMV and UCP2 overexpressed cells. β-actin served as loading control. (ii) Fru-2,6-P_2_ concentration **(C)** and PFK levels **(D)** were measured using control pCMV and UCP2 overexpressed cells. n=4 per group, data are presented as the mean ± SD. ^*^, p<0.05 when compared with its own control/DMSO group (n=4 in each group); ^#^, p<0.05 when compared with the control/TPA group.

During our previous skin carcinogenesis study using UCP2 KO mice, phosphorylated PFKFB2 was found to be upregulated in skin tumor tissues of the wild-type mice but not the UCP2 knockout mice [28], suggesting that UCP2 may positively regulate PFKFB2 expression. PFKFB2 is a bifunctional enzyme which controls the levels of Fru-2,6-P_2_, a power activator of PFK-1, which is a critical regulator of glycolytic flux. Therefore, an increase in the level of Fru-2,6-P_2_ could lead to a higher glycolytic rate, which has been detected in certain cancer cells [32–34]. Therefore, to investigate whether UCP2 overexpression positively correlates with PFKFB2 upregulation, we examined the protein expression of PFKFB2 and its phosphorylated form (Ser483, phosphorylation of PFKFB2 is associated with an increase in its kinase activity) using UCP2 overexpressed and control pCMV cells. As shown in Figure 2A & 2B, PFKFB2 and pPFKFB2 expression was indeed increased in UCP2 overexpressed cells and TPA treatment further induced PFKFB2 expression. We next measured the levels of Fru-2,6-P_2_ and the results (Figure 2C) demonstrated that Fru-2,6-P_2_ levels were elevated in the UCP2 overexpressed cells and in response to the TPA treatment. In addition, to study whether the increase in the levels of Fru-2,6-P_2_ correlates with an increase in PFK-1, we measured the activity of this enzyme. As shown in the Figure 2D, PFK-1 activity was significantly higher in the UCP2 overexpression cells, and TPA treatment further enhanced this increase.

### PFKFB2 upregulation contributed to metabolic switch towards glycolysis in UCP2 overexpressed cells

**Figure 3:**
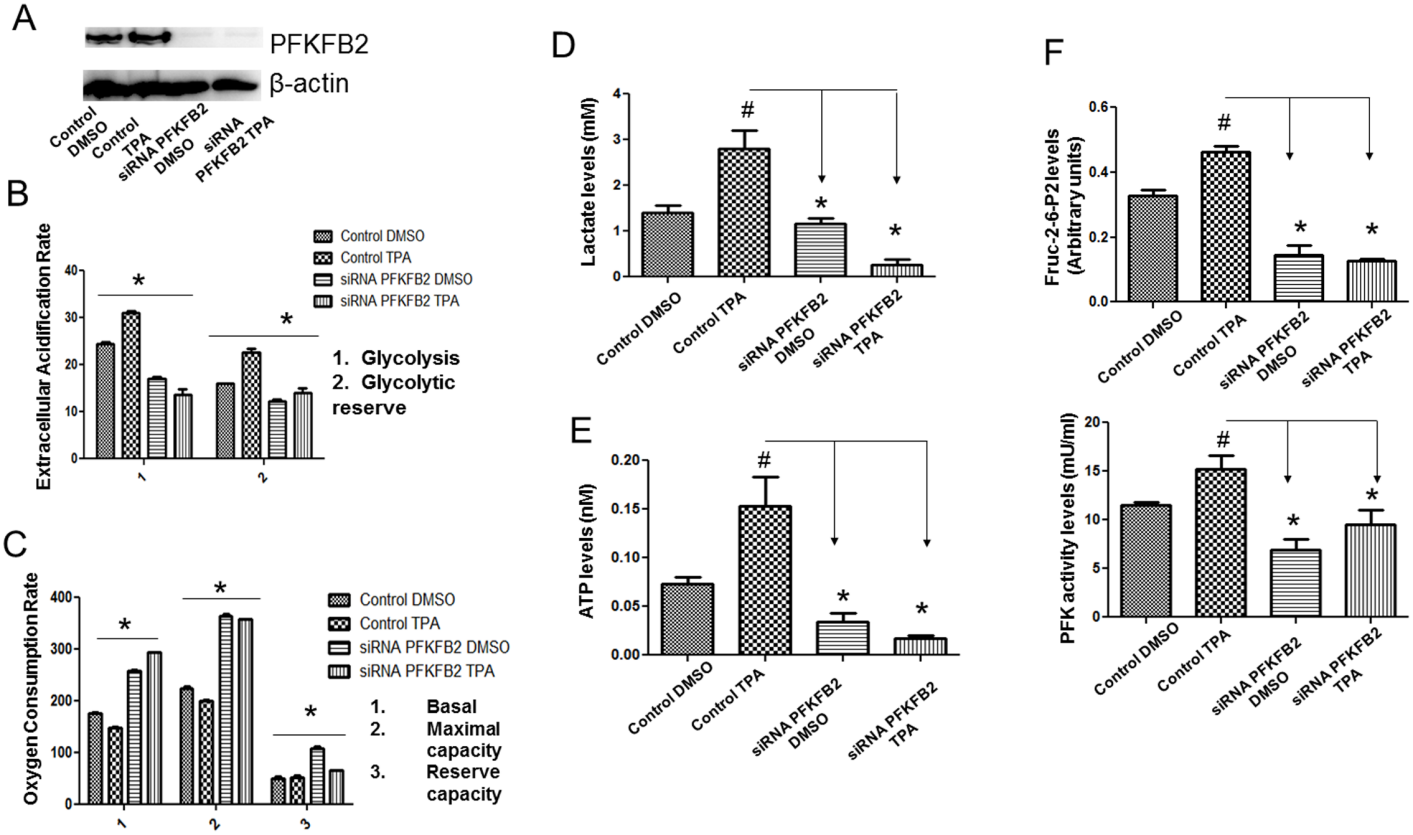
Inhibition of PFKFB2 expression dampens the shift to glycolysis in UCP2 overexpressed cells (i) OCR, an indicator of mitochondrial oxidative phosphorylation **(C)** and ECAR, an indicator of glycolysis **(B)**, were measured using control siRNA and siRNA PFKFB2 knockdown cells **(A)**. Inhibition of PFKFB2 expression resulted in decreased glycolytic rate, whereas, an increased OCR suggested that PFKFB2 promotes cellular adaptation towards enhanced glycolysis. Results were an average of three independent experiments. Statistical analysis was performed using two-way ANOVA with test. ^*^p<0.001 (N=3). (ii) Knockdown of PFKFB2 expression resulted in decreased lactate levels **(D)**, decreased cellular ATP levels **(E)**, and reduced Fru-2,6-P_2_ concentration and PFK levels **(F)** in UCP2 overexpressed cells, suggesting that PFKFB2 allows UCP2 overexpressed cells to maintain higher glycolysis leading to enhanced lactate and ATP production. n=4 per group, data are presented as the mean ± SD. ^*^, p<0.05 when compared with its own control/DMSO group (n=3 in each group); ^#^, p<0.05 when compared with the control/TPA group.

To study whether PFKFB2 mediates UCP2's role in regulating glycolysis, we used a siRNA approach to knockdown PFKFB2 and measured OCR and ECAR rates using the knockdown cells. Briefly, UCP2 overexpressed cells were transfected with PFKFB2 siRNA or the control siRNA, and OCR and ECAR were measured using the Seahorse Bioscience XF Analyzer as described above. As shown in Figure 3, a reduction in PFKFB2 expression (Figure 3A) resulted in decreased glycolysis (Figure 3B), whereas it enhanced mitochondrial respiration (Figure 3C) in the UCP2 overexpressed cells. Reduction in glycolysis was concomitant with the fall in lactate production (Figure 3D) as observed in the PFKFB2 knockdown cells. In addition, the reduction in the ATP levels (Figure 3E) in the UCP2 overexpressed cells are suggestive of dampened glycolysis. These data indicate the dependence of UCP2 overexpressed cells on glycolysis for ATP production (the Warburg effect). Collectively, these results thus demonstrate that PFKFB2 mediates UCP2's role in regulating glycolysis. Moreover, to study whether PFKFB2 is required for the increase in Fru-2,6-P_2_ concentration and PFK-1 activity, we measured their levels using control cells and PFKFB2 siRNA knockdown cells. Indeed, inhibition of PFKFB2 expression reduced the levels of Fru-2,6-P_2_ and PFK-1 activity in UCP2 overexpressed cells (Figure 3F, top and bottom panels). Therefore, these data suggest that the increases in Fru-2,6-P_2_ concentrations and PFK-1 activity are due to the upregulation of PFKFB2 expression in the UCP2 overexpressed cells.

### Knockdown of PKFKB2 suppressed skin cell transformation in UCP2 overexpressed cells

**Figure 4:**
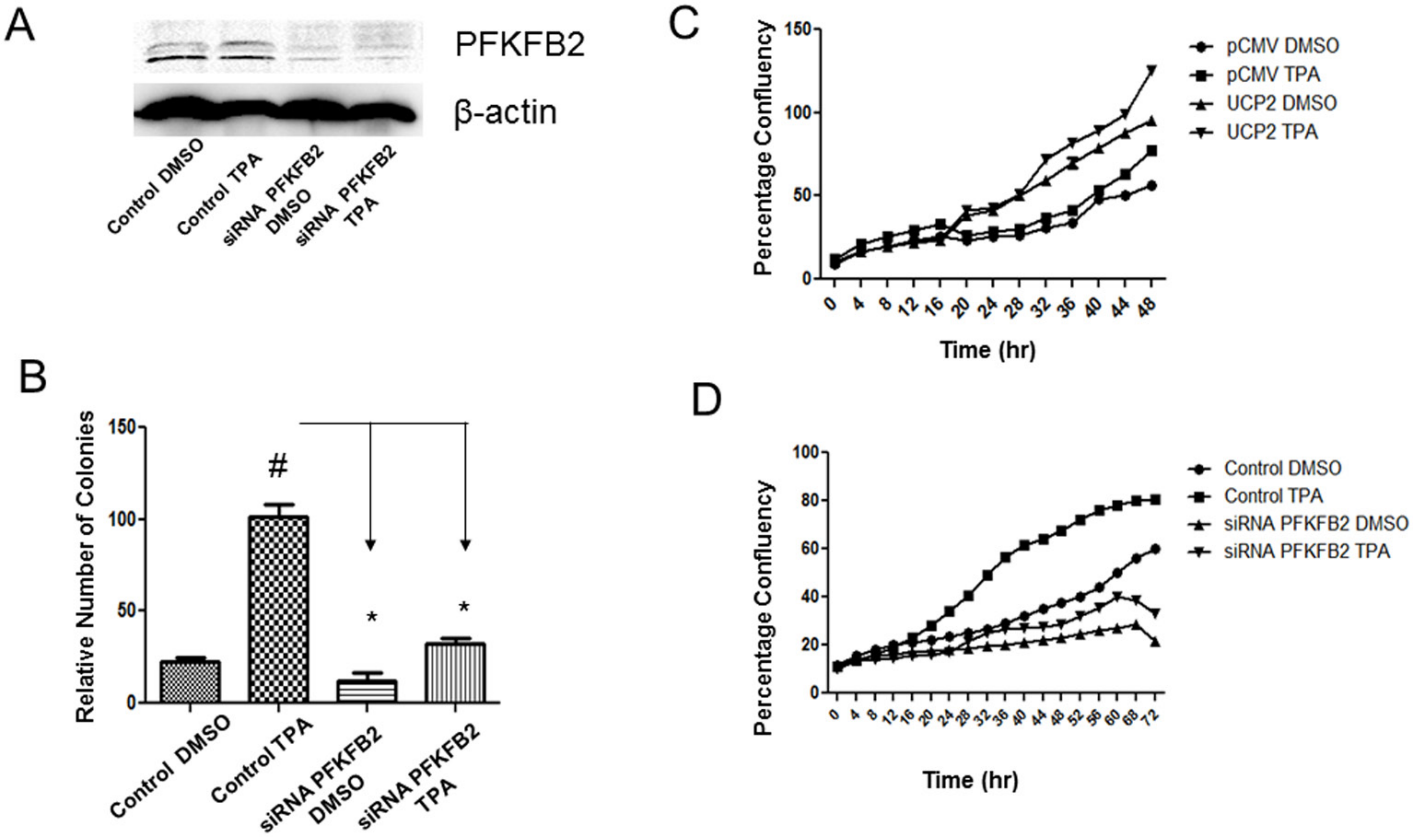
siRNA knockdown of PKFKB2 suppressed skin tumorigenesis in UCP2 overexpressed cells (i) Western blot analysis confirming PFKFB2 knockdown **(A)**. Soft agar assays were performed to detect anchorage-independent growth using control siRNA and siRNA PFKFB2 cells were used in the assay **(B)**. Ctrl: vehicle (DMSO) treatment; TPA, 5 nM. ^*^, p<0.05 when compared with its own control/DMSO group (n=3 in each group); ^#^, p<0.05 when compared with the control/TPA group. (ii) Cell proliferation assay using the IncuCyte zoom live-cell analysis was performed in control pCMV and UCP2 overexpressed cells **(C)**, and control siRNA and siRNA PFKFB2 cells **(D)**. 1,500 cells/well were seeded in the 96 well-plate and incubated overnight in the IncuCyte incubator. Cells were treated with Ctrl: vehicle (DMSO) treatment; TPA, 5 nM. Cell confluency was measured using the IncuCyte zoom software.

Since PFKFB2 is highly expressed in several cancers and its expression is positively correlated with carcinogenesis [31–34], we used siRNA interference to study whether inhibition of PFKFB2 reduces tumorigenicity of UCP2 overexpressed cells. We first transfected UCP2 overexpressed cells with PFKFB2 siRNA or the control siRNA. As shown in the Figure 4A, successful knockdown of PFKFB2 was observed. Next, we performed anchorage independent growth assays, and our results demonstrated that colony numbers from PFKFB2 knockdown cells were markedly lower than that from the control siRNA cell (Figure 4B). Next, to determine the effect of PFKFB2 knockdown on cell proliferation, we used the IncuCyte Zoom to monitor the cell proliferation rate. As shown in Figure 4C, UCP2 overexpressed cells have an enhanced proliferation rate and TPA treatment further enhances cell growth compared to that of the control pCMV cells. On measuring the growth rate using control UCP2 cells and PFKFB2 knockdown UCP2 cells, the result (Figure 4D) showed that knockdown of PFKFB2 significantly suppressed cell growth rate. These data indicate a possible role of PFKFB2 in the regulation of cell growth and cell transformation mediated by UCP2 overexpression.

### Involvement of the MAPK pathway in UCP2 overexpressed cells

**Figure 5:**
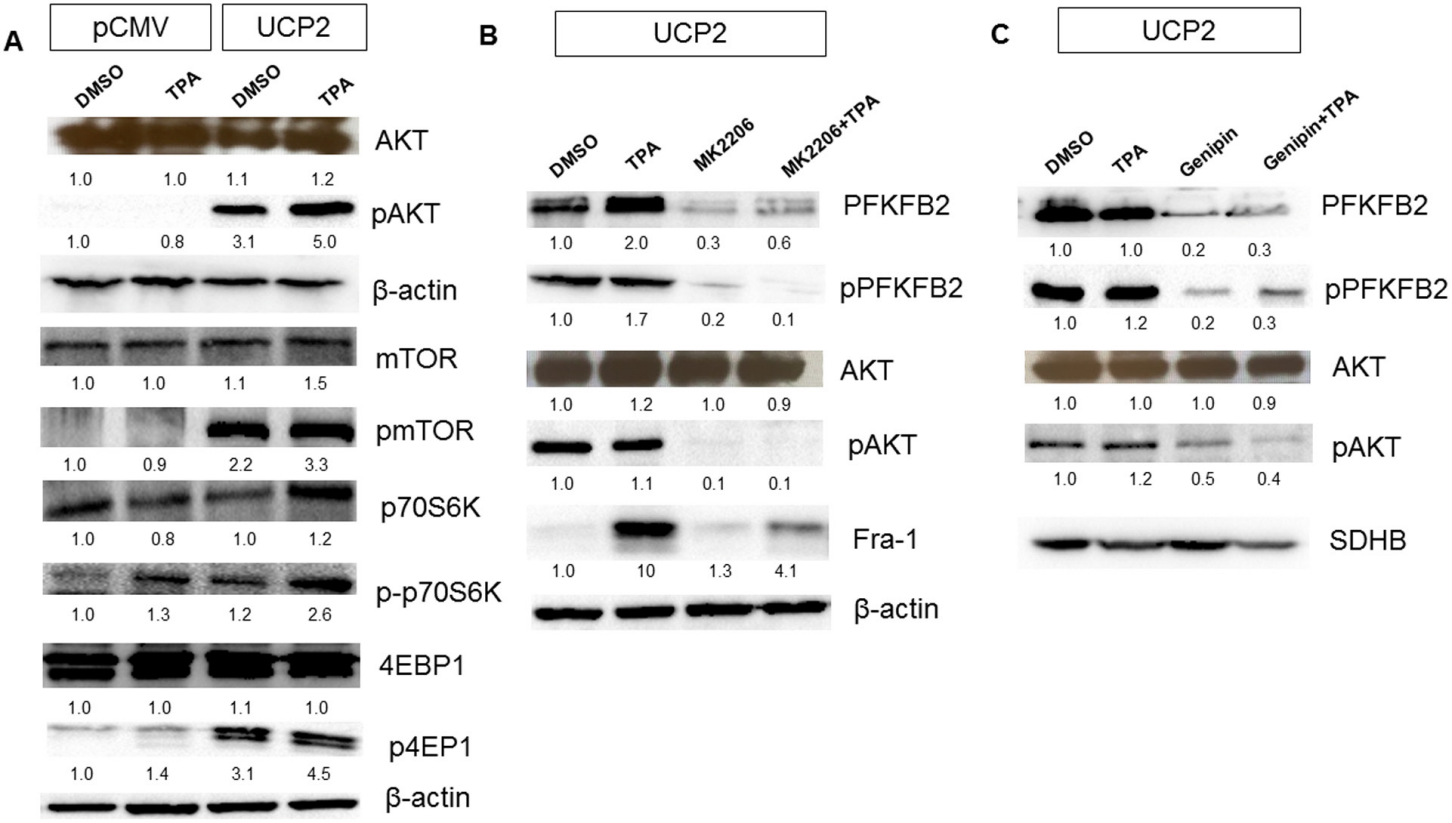
UCP2 induces PFKFB2 expression in AKT-dependent manner (i) Western blot analysis of AKT, pAKT, mTORC1, p-mTOR, p70S6K, p-p70S6K, 4EBP1, and p-4EBP1 in UCP2 overexpressed and control pCMV cells **(A)**. (i) Western blots analysis of PFKFB2, pPFKFB2, AKT, and pAKT levels from UCP2 overexpressed cells treated with MK2206 **(B)**. (iii) Western blots analysis of PFKFB2, pPFKFB2, AKT, and pAKT levels from UCP2 overexpressed cells treated with genipin **(C)**.

Recently, it has been demonstrated that MAPK, particularly AKT, is involved in the activation of PFKFB2 [37]. Various studies have demonstrated that MAPK promotes cancer cell proliferation and differentiation [38–42] and it is no surprise that these pathways are overexpressed in cancers. To further assess the role of AKT pathway in the activation PFKFB2, we measured the protein expression levels of AKT and one of its downstream targets, mTOR. As shown in Figure 5A, the expression levels of AKT, pAKT, mTORC1, p-mTORC1, and its downstream regulators: p70S6K (ribosomal p70S6 kinase) and 4E-BP (eukaryotic initiation factor 4E-binding protein) were upregulated in UCP2 overexpressed cells compared to the control pCMV cells, implying that AKT is activated by UCP2 overexpression.

To further assess the role of AKT in the increase in PFKFB2, we used the specific inhibitor to AKT MK2206 [43]. As shown in Figure 5B, MK2206 treatment suppressed PFKFB2 expression and phosphorylation (Ser483). Taken together, these results indicate a key role for AKT in the activation of PFKFB2.

Next, we studied whether applying the UCP2 inhibitor genipin could suppress AKT and PFKFB2 activation. Genipin, a natural water-soluble chemical extracted from *gardenia fruits* has been extensively used in Chinese herbal medicines [44–45]. Numerous studies show genipin's safety and efficacy for use in patients with diabetes, periodontitis, cataract, hepatic dysfunction, and cancer [46–49]. Genipin is known to be highly selective and specific to UCP2's inhibition and has been shown to sensitize drug-resistant cancer cells by inhibiting the actions of UCP2 [48]. Our earlier studies indicate that genipin at the concentration of 10 μM is sufficient to inhibit the expression of UCP2 and suppresses the 3D growth of UCP2 overexpressed cells *in vitro* (data unpublished, under review). We treated UCP2 overexpressed cells with genipin and then examined AKT and PFKFB2 activation. Clearly, as shown in Figure 5C, genipin suppressed the activation of both AKT and PFKFB2. All of these results suggest that UCP2 may play a crucial role in activating PFKFB2 via the activation of AKT.

## DISCUSSION

UCP2, an anion/ion transporter present in the inner mitochondrial membrane, is closely associated with mitochondrial redox signaling, ROS regulation, apoptosis, cell growth, and survival [50–51]. In human cancers, UCP2 is overexpressed in a number of aggressive cancers including prostate, kidney, thyroid, skin, etc. [52–53, 15, 16, 28]. UCP2 transfers anions from the inner to the outer mitochondrial membrane and facilitates the transfer of protons back into the inner mitochondrial membrane, leading to the reduction of mitochondrial superoxide production. Hence, UCP2 overexpression is thought to confer a growth advantage for cancer cells. In addition, highly expressed UCP2 could confer chemoresistance and inhibition of UCP2 expression sensitizes cancer cells to chemotherapy [54]. Recently, UCP2 has also been demonstrated to transport TCA cycle C4 metabolites out of the mitochondria [27]. The consideration of UCP2 as a metabolite transporter has led to a more encompassing idea that UCP2 may contribute to cancer metabolism and malignant transformation [27, 55].

A mounting body of evidence has continued to unequivocally demonstrate that cancer cells have altered metabolism [1]. This feature of metabolic reprogramming of cancer cells is not new and dates back to the early 1920’s. One of the hallmark features of metabolic reprogramming in cancer cells is the enhanced glycolysis leading to lactate production even in the presence of oxygen, as proposed by Otto Warburg [1, 3]. The metabolite transporter activity of UCP2 provides a strong rationale for the notion that highly expressed UCP2 in cancer cells contributes to the Warburg effect [56]. However, how exactly glycolysis is affected by UCP2 is not known.

Based on the mouse skin carcinogenesis study [28], PFKFB2 in the glycolysis pathway was identified as a potential target for UCP2. In the same study, UCP2 contributed to the increase of the skin tissue levels of pyruvate and malate [28]. To reveal the mechanism of how UCP2 may regulate PFKFB2 activity, our results used the JB6 skin cell transformation model to provide direct evidence that UCP2 overexpression suppresses mitochondrial oxidative phosphorylation while augmenting glycolysis, leading to increased lactate production. UCP2 overexpression contributes to enhanced glycolysis by activating PFKFB2. In contrast, siRNA mediated inhibition of PFKFB2 causes a marked decrease in glycolysis, cell proliferation, and cell transformation in UCP2 overexpressed cells.

Future studies will be needed to validate how TCA cycle intermediates may regulate PFKFB2 activity? Since the AKT/mTOR pathway can sense the changes in nutrients [57–58], which becomes the candidate. Our studies demonstrate that AKT indeed is required for the activation of PFKFB2 in UCP2 overexpressed cells. Since the alterations in cellular metabolism and the metabolic switch are relevant to many tumor cells, we believe that PFKFB2 could potentially be an interesting candidate in the association of tumorigenesis and metabolism in UCP2 highly expressed cancers.

**Figure:**
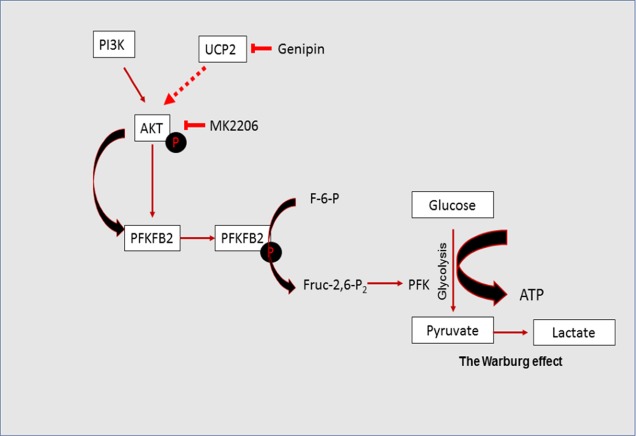
UCP2 directs the metabolic switch towards glycolysis by activating PFKFB2

In this study, we provide new evidence that UCP2 plays a critical role in the regulation of the metabolic switch during skin tumorigenesis. UCP2 appears to be an interesting crosslink between cellular bioenergetics and tumorigenesis. However, whether UCP2 upregulation is the cause or the effect of tumorigenesis, whether UCP2's transporter activity is directly or indirectly responsible for the activation of PFKFB2, and whether UCP2-induced PFKFB2 directly contributes to malignant transformation need to be addressed in future studies. In conclusion, our studies demonstrated that: (i) UCP2 overexpression positively correlates with PFKFB2 activity; (ii) UCP2-induced PFKFB2 upregulation leads to enhanced glycolysis; (iii) AKT may mediate UCP2-induced PFKFB2 activation; and (iv) targeting UCP2 and/or PFKFB2 may serve as a novel therapeutic approach for UCP2 highly expressed cancers. Together, these observations suggest that UCP2 is an important regulator of mitochondrial and cellular bioenergetics.

## MATERIALS AND METHODS

### Cell culture

Murine skin epidermal JB6 P+ cells (purchased from American Type Culture Collection) were used in the study. Human UCP2 and empty pCMV6 vector transfected JB6 clones have been generated and characterized (manuscript under review). These cells were grown in EMEM media supplemented with 4% heat-inactivated fetal bovine serum (FBS), 2 mM glutamine, 100 U/mL penicillin, 100 μg/mL streptomycin, and 200 μg/μL G418. The tumor promoter 12-*O*-tetradecanoylphorbol 13-acetate (TPA, purchased from Sigma) was prepared as 1 mM stock solution in DMSO. The stock solutions were diluted in culture media to a final concentration of 5 nM.

### Transfection of siRNA

UCP2 overexpressed JB6 cells were incubated in a six-well tissue culture plate 24 hours before transfection. The next day, with the cells reaching 60%–80% confluency, the control (scramble) siRNA (Catalog number AM4611) or target-specific PFKFB2 siRNA (Catalog number AM16708, both purchased from Life Technologies), was mixed with the siRNA transfection reagent according to manufacturer's recommendation and added to the cells. After incubation for 6-hours at 37°C, the transfection mix was replaced with normal growth medium. The expression levels of PFKFB2 were examined by Western blot analysis with specific antibodies (Catalog Number TA314335, purchased from OriGene Technologies).

### Western blot analysis

Cells were collected, and whole cell lysate was prepared as previously described [59]. Cell lysates were mixed with 4 × sodium dodecyl sulfate (SDS) sample buffer and denatured by heating at 98°C for 5 minutes. Equal amounts of denatured proteins were separated by 10% SDS–polyacrylamide gel electrophoresis and then transferred onto polyvinylidene fluoride membranes. After blocking for 2 hours with phosphate-buffered saline (PBS) containing 0.1% Tween 20 and 5% skim milk, the blots were incubated with correspondent primary antibodies. After the membranes were washed three times with PBS with 0.1% Tween 20, they were then incubated with horseradish peroxidase-conjugated secondary antibodies. All bands were detected using an ECL Western blot kit. Antibodies for p-PFKFB2 (#13064), mTOR (#2983), p-mTOR (#2971), 4EBP1 (#9452), p-4EBP1 (#13396), p70S6K (#34499), p-p70S6K (#9204), AKT (#2920), and p-AKT (#4060) were purchased from Cell Signaling Technologies. SDHB or β-actin (SC-25851 and SC-47778 respectively, both purchased from Santa Cruz Biotechnology) was used as the loading control.

### Colony formation assay

Soft agar colony formation assay was used to determine the effect of PFKFB2 downregulation on anchorage-independent growth. Briefly, 0.5% agar mix (40 ml melted 1.25% agar solution, 40 ml 2×EMEM, 10 ml FBS, 10 ml PBS, 1 ml glutamine, 50 μl penicillin, and streptomycin) was prepared and kept in a 50°C water bath. Bottom agar was prepared by adding DMSO or TPA to the 0.5% agar mix. Top agar was prepared by diluting 1 fraction of 1×10^5^ cells/ml single cell suspension with 2 fractions of 0.5% agar mix plus DMSO/TPA treatments. Bottom agar (2.5 ml) and top agar (0.75 ml) were laid into each well of the 6-well plates. Plates were incubated in a humidified 37°C, 5% CO_2_ incubator for 2 weeks. Cells were then stained with 0.25 mg/ml neutral red overnight, and the colonies were counted and plotted.

### Detection of lactate levels

The levels of lactate were determined using the Lactate Assay Kit (BioVison, K607-100) following the instructions provided by the manufacturer. Total cell lysate, prepared in PBS with proteinase inhibitors, was diluted to 2 μg/μl in PBS and deproteinized by passing through a 10 kD cut-off membrane (VWR, 82031-348). For each sample, 50 μl of the whole cell lysate filtrate were used.

### Detection of intracellular ATP levels

Cellular ATP content was analyzed using the ATP Assay Kit (BioVision, K354-100), according to the manufacturer's protocol. Deproteinized total cell lysates were used for the assay.

### Measurement of fructose-2,6-bisphosphate levels

Fructose-2,6-bisphosphate levels were determined based on the activation of pyrophosphate-dependent PFK1, as previously described [60–61]. Cells were pelleted by low-speed centrifugation and resuspended in a solution containing 10-100 volumes of 50 mM NaOH. The solution was then heated at 80°C for 5 minutes before being placed on ice, and the solution was neutralized with cold acetic acid in 20 mM HEPES buffer. Then, 0.5 mM pyrophosphate was added and absorbance was measured at OD 340 nm. The F-2,6BP concentration was normalized to total protein content.

### Cell growth using IncuCyte zoom life cell imagine system

Three thousand cells per well were seeded in 96-well plates and incubated overnight in the IncuCyte Essens Bioscience incubator (Birmingham, U.K.). Live cell images were collected every 4 hours. Proliferation rates based on cell confluence were determined by live cell imaging using the IncuCyte software.

### Mitochondrial bioenergetics measurements

Oxygen consumption was determined using the Seahorse Extracellular Flux (XF-24) analyzer (Seahorse Bioscience, Chicopee, MA). The XF-24 measures the concentration of oxygen and free protons in the medium above a monolayer of cells in real time. Thus, the rates of oxygen consumption and proton production can be measured across several samples at a time. To allow comparison between experiments, data are presented as oxygen consumption rate (OCR) in pMoles/min/10^4^ cells and the extracellular acidification rate (ECAR) in mpH/min/10^4^ cells. Cells were seeded at 50,000 cells per well into gelatin-coated Seahorse Bioscience XF 24-well plates, cultured in the presence or absence of 2 g/L D-glucose, and then centrifuged to adhere to the bottom of the wells.

Oxygen consumption rate (OCR) was measured four times and plotted as a function of cells under the basal condition followed by the sequential addition of oligomycin (1 μg/ml), an inhibitor of mitochondrial ATP-synthase, to estimate the OCR coupled to ATP synthesis and represented as ATP-linked. The residual OCR minus the non-mitochondrial OCR can be attributed to proton leak; the uncoupler FCCP (1 μM) was added to determine the maximal OCR; antimycin (1 μM), an inhibitor of mitochondrial respiration, was added to determine non-mitochondrial sources of oxygen consumption. The ATP-linked OCR was calculated as the difference between the basal OCR and the OCR measured after the addition of oligomycin. The OCR maximal capacity was the direct rate measured after the addition of FCCP. Reserve capacity is a measure of the amount of ATP that can be produced under energetic demand and was calculated as the difference between the maximum and the basal rate of respiration. The OCR values were normalized to total protein content in the corresponding wells and expressed as pmol/min/mg protein.

For extracellular acidification rate (ECAR) measurements, cells were washed and changed to assay media lacking glucose. Basal ECAR was measured four times and plotted as a function of cells under the basal condition followed by the sequential addition of glucose (25 mM), oligomycin (1 μg/ml) and 2-deoxyglucose (25 mM), an inhibitor for the hexokinase. The rate of glycolysis was determined by the difference between the basal ECAR and the ECAR after the addition of glucose. Glycolytic reserve was determined by subtracting the ECAR following the addition of oligomycin from the ECAR following the addition of glucose. Differences between treatment groups were calculated using two-way analysis of variance (ANOVA).

### Statistical analysis

Each assay was performed at least three times. Statistical software SAS 9.4 (SAS Institute Inc.) was used for all data analysis. The data were expressed as mean ± SD. ^*^p < 0.05 were considered to be statistically significant.

## Abbreviations

ECAR: extracellular acidification rate
FBS: fetal bovine serum
FCCP: p-trifluoromethoxy carbonyl cyanide phenyl hydrazine
Fru-2,6-P_2_: 2,6-bisphosphate
OCR: oxygen consumption rate
PBS: phosphate-buffered saline
PFK-1: 6-phosphofructo-1-kinase
PFKFB2: phosphofructokinase 2/fructose-2,6-bisphosphatase 2
UCP2: uncoupling protein 2
SDS: sodium dodecyl sulfate
TPA: 12-*O*-tetradecanoylphorbol 13-acetate

## References

[1] Hanahan D, Weinberg RA Hallmarks of cancer: the next generation. cell.

[2] Phan LM, Yeung SC, Lee MH Cancer metabolic reprogramming: importance, main features, and potentials for precise targeted anti-cancer therapies. Cancer biology & medicine.

[3] Warburg O On the origin of cancer cells. Science.

[4] Ward PS, Thompson CB Metabolic reprogramming: a cancer hallmark even warburg did not anticipate. Cancer cell.

[5] Kim JW, Dang CV Cancer's molecular sweet tooth and the Warburg effect. Cancer research.

[6] Vander Heiden MG, Cantley LC, Thompson CB Understanding the Warburg effect: the metabolic requirements of cell proliferation. science.

[7] Gatenby RA, Gillies RJ Why do cancers have high aerobic glycolysis?. Nature Reviews Cancer.

[8] Hsu PP, Sabatini DM Cancer cell metabolism: Warburg and beyond. Cell.

[9] Liberti MV, Locasale JW The Warburg effect: how does it benefit cancer cells? Trends in biochemical sciences.

[10] Hirschey MD, DeBerardinis RJ, Diehl AM, Drew JE, Frezza C, Green MF, Jones LW, Ko YH, Le A Lea MA, Locasale JW (2015). Dysregulated metabolism contributes to oncogenesis. InSeminars in cancer biology.

[11] Seyfried TN, Shelton LM Cancer as a metabolic disease. Nutrition & metabolism.

[12] Pelicano H, Martin DS, Xu RA, Huang P Glycolysis inhibition for anticancer treatment. Oncogene.

[13] Lunt SY, Vander Heiden MG Aerobic glycolysis: meeting the metabolic requirements of cell proliferation. Annual review of cell and developmental biology.

[14] Dang CV Links between metabolism and cancer. Genes & development.

[15] Robbins D, Zhao Y New aspects of mitochondrial uncoupling proteins (UCPs) and their roles in tumorigenesis. International journal of molecular sciences.

[16] Sreedhar A, Zhao Y (2017). Uncoupling protein 2 and metabolic diseases. Mitochondrion.

[17] Ayyasamy V, Owens KM, Desouki MM, Liang P, Bakin A, Thangaraj K, Buchsbaum DJ, LoBuglio AF, Singh KK Cellular model of Warburg effect identifies tumor promoting function of UCP2 in breast cancer and its suppression by genipin. PloS one.

[18] Cordani M, Butera G, Pacchiana R, Donadelli M (2017). The antioxidant mitochondrial protein UCP2 promotes cancer development connecting the Warburg effect and autophagy. Translational Medicine Reports.

[19] Derdak Z, Mark NM, Beldi G, Robson SC, Wands JR, Baffy G The mitochondrial uncoupling protein-2 promotes chemoresistance in cancer cells. Cancer research.

[20] Li W, Nichols K, Nathan CA, Zhao Y Mitochondrial uncoupling protein 2 is up-regulated in human head and neck, skin, pancreatic, and prostate tumors. Cancer Biomarkers.

[21] Horimoto M, Resnick MB, Konkin TA, Routhier J, Wands JR, Baffy G Expression of uncoupling protein-2 in human colon cancer. Clinical Cancer Research.

[22] Pons DG, Nadal-Serrano M, Torrens-Mas M, Valle A, Oliver J, Roca P UCP2 inhibition sensitizes breast cancer cells to therapeutic agents by increasing oxidative stress. Free Radical Biology and Medicine.

[23] Nègre-Salvayre A, Hirtz C, Carrera G, Cazenave R, Troly M, Salvayre R, Pénicaud L, Casteilla L A role for uncoupling protein-2 as a regulator of mitochondrial hydrogen peroxide generation. The FASEB Journal.

[24] Arsenijevic D, Onuma H, Pecqueur C, Raimbault S, Manning BS, Miroux B, Couplan E, Alves-Guerra MC, Goubern M, Surwit R, Bouillaud F Disruption of the uncoupling protein-2 gene in mice reveals a role in immunity and reactive oxygen species production. Nature genetics.

[25] Zhang CY, Baffy G, Perret P, Krauss S, Peroni O, Grujic D, Hagen T, Vidal-Puig AJ, Boss O, Kim YB, Zheng XX Uncoupling protein-2 negatively regulates insulin secretion and is a major link between obesity, β cell dysfunction, and type 2 diabetes. Cell.

[26] Mattiasson G, Sullivan PG The emerging functions of UCP2 in health, disease, and therapeutics. Antioxidants & redox signaling.

[27] Vozza A, Parisi G, De Leonardis F, Lasorsa FM, Castegna A, Amorese D, Marmo R, Calcagnile VM, Palmieri L, Ricquier D, Paradies E UCP2 transports C4 metabolites out of mitochondria, regulating glucose and glutamine oxidation.

[28] Li W, Zhang C, Jackson K, Shen X, Jin R, Li G, Kevil CG, Gu X, Shi R, Zhao Y UCP2 knockout suppresses mouse skin carcinogenesis. Cancer Prevention Research.

[29] Pilkis SJ, Claus TH, Kurland IJ, Lange AJ 6-Phosphofructo-2-kinase/fructose-2, 6-bisphosphatase: a metabolic signaling enzyme. Annual review of biochemistry.

[30] Yalcin A, Telang S, Clem B, Chesney J Regulation of glucose metabolism by 6-phosphofructo-2-kinase/fructose-2, 6-bisphosphatases in cancer. Experimental and molecular pathology.

[31] Okar DA, Lange AJ, À Manzano, Navarro-Sabatè A, Riera L, Bartrons R PFK-1/FBPase-2: maker and breaker of the essential biofactor fructose-2, 6-bisphosphate. Trends in biochemical sciences.

[32] Minchenko OH, Ochiai A, Opentanova IL, Ogura T, Minchenko DO, Caro J, Komisarenko SV, Esumi H Overexpression of 6-phosphofructo-2-kinase/fructose-2, 6-bisphosphatase-4 in the human breast and colon malignant tumors. Biochimie.

[33] Minchenko OH, Ogura T, Opentanova IL, Minchenko DO, Ochiai A, Caro J, Komisarenko SV, Esumi H 6-Phosphofructo-2-kinase/fructose-2, 6-bisphosphatase gene family overexpression in human lung tumor. Ukr Biokhim Zh.

[34] Chesney J 6-phosphofructo-2-kinase/fructose-2, 6-bisphosphatase and tumor cell glycolysis. Current Opinion in Clinical Nutrition & Metabolic Care.

[35] Ferrick DA, Neilson A, Beeson C Advances in measuring cellular bioenergetics using extracellular flux. Drug discovery today.

[36] Dranka BP, Benavides GA, Diers AR, Giordano S, Zelickson BR, Reily C, Zou L, Chatham JC, Hill BG, Zhang J, Landar A Assessing bioenergetic function in response to oxidative stress by metabolic profiling. Free Radical Biology and Medicine.

[37] Novellasdemunt L, Tato I, Navarro-Sabate A, Ruiz-Meana M, Méndez-Lucas A, Perales JC, Garcia-Dorado D, Ventura F, Bartrons R, Rosa JL Akt-dependent activation of the heart 6-phosphofructo-2-kinase/fructose-2, 6-bisphosphatase (PFKFB2) isoenzyme by amino acids. Journal of Biological Chemistry.

[38] Dhillon AS, Hagan S, Rath O, Kolch W MAP kinase signalling pathways in cancer. Oncogene.

[39] McCubrey JA, Steelman LS, Chappell WH, Abrams SL, Wong EW, Chang F, Lehmann B, Terrian DM, Milella M, Tafuri A, Stivala F Roles of the Raf/MEK/ERK pathway in cell growth, malignant transformation and drug resistance. Biochimica et Biophysica Acta (BBA)-Molecular Cell Research.

[40] Zhang W, Liu HT MAPK signal pathways in the regulation of cell proliferation in mammalian cells. Cell research.

[41] Roberts PJ, Der CJ Targeting the Raf-MEK-ERK mitogen-activated protein kinase cascade for the treatment of cancer. Oncogene.

[42] Wan PT, Garnett MJ, Roe SM, Lee S, Niculescu-Duvaz D, Good VM, Project CG, Jones CM, Marshall CJ, Springer CJ, Barford D Mechanism of activation of the RAF-ERK signaling pathway by oncogenic mutations of B-RAF. Cell.

[43] Hirai H, Sootome H, Nakatsuru Y, Miyama K, Taguchi S, Tsujioka K, Ueno Y, Hatch H, Majumder PK, Pan BS, Kotani H MK-2206, an allosteric Akt inhibitor, enhances antitumor efficacy by standard chemotherapeutic agents or molecular targeted drugs in vitro and in vivo. Molecular cancer therapeutics.

[44] Yamamoto M, Miura N, Ohtake N, Amagaya S, Ishige A, Sasaki H, Komatsu Y, Fukuda K, Ito T, Terasawa K Genipin, a metabolite derived from the herbal medicine Inchin-ko-to, and suppression of Fas-induced lethal liver apoptosis in mice. Gastroenterology.

[45] Muzzarelli RA Genipin-crosslinked chitosan hydrogels as biomedical and pharmaceutical aids. Carbohydrate Polymers.

[46] Zhang CY, Parton LE, Ye CP, Krauss S, Shen R, Lin CT, Porco JA, Lowell BB Genipin inhibits UCP2-mediated proton leak and acutely reverses obesity-and high glucose-induced β cell dysfunction in isolated pancreatic islets. Cell metabolism.

[47] Sun GD, Li CY, Cui WP, Guo QY, Dong CQ, Zou HB, Liu SJ, Dong WP, Miao LN (2015;2016). Review of herbal traditional chinese medicine for the treatment of diabetic nephropathy. Journal of diabetes research.

[48] Mailloux RJ, Adjeitey CN, Harper ME Genipin-induced inhibition of uncoupling protein-2 sensitizes drug-resistant cancer cells to cytotoxic agents. PloS one.

[49] Ayyasamy V, Owens KM, Desouki MM, Liang P, Bakin A, Thangaraj K, Buchsbaum DJ, LoBuglio AF, Singh KK Cellular model of Warburg effect identifies tumor promoting function of UCP2 in breast cancer and its suppression by genipin. PloS one.

[50] Baffy G Uncoupling protein-2 and cancer. Mitochondrion.

[51] Nègre-Salvayre A, Hirtz C, Carrera G, Cazenave R, Troly M, Salvayre R, Pénicaud L, Casteilla L A role for uncoupling protein-2 as a regulator of mitochondrial hydrogen peroxide generation. The FASEB Journal.

[52] Horimoto M, Resnick MB, Konkin TA, Routhier J, Wands JR, Baffy G Expression of uncoupling protein-2 in human colon cancer. Clinical Cancer Research.

[53] Sayeed A, Meng Z, Luciani G, Chen LC, Bennington JL, Dairkee SH Negative regulation of UCP2 by TGFβ signaling characterizes low and intermediate-grade primary breast cancer. Cell death & disease.

[54] Derdak Z, Mark NM, Beldi G, Robson SC, Wands JR, Baffy G The mitochondrial uncoupling protein-2 promotes chemoresistance in cancer cells. Cancer research.

[55] Pecqueur C, Alves-Guerra C, Ricquier D, Bouillaud F UCP2, a metabolic sensor coupling glucose oxidation to mitochondrial metabolism?. IUBMB life.

[56] Samudio I, Fiegl M, Andreeff M Mitochondrial uncoupling and the Warburg effect: molecular basis for the reprogramming of cancer cell metabolism. Cancer research.

[57] Sengupta S, Peterson TR, Sabatini DM Regulation of the mTOR complex 1 pathway by nutrients, growth factors, and stress. Molecular cell.

[58] Düvel K, Yecies JL, Menon S, Raman P, Lipovsky AI, Souza AL, Triantafellow E, Ma Q, Gorski R, Cleaver S, Vander Heiden MG Activation of a metabolic gene regulatory network downstream of mTOR complex 1. Molecular cell.

[59] Robbins D, Ponville J, Morris K, Zhao Y Involvement of PTEN in TPA-mediated p53-activation in mouse skin epidermal JB6 cells. FEBS letters.

[60] Schaftingen E, Lederer B, Bartrons R, Hers HG (1982). A Kinetic Study of Pyrophosphate: Fructose-6-Phosphate Phosphotransferase from Potato Tubers. The FEBS Journal.

[61] Manzano A, Rosa JL, Ventura F, Perez JX, Nadal M, Estivill X, Ambrosio S, Gil J, Bartrons R Molecular cloning, expression, and chromosomal localization of a ubiquitously expressed human 6-phosphofructo-2-kinase/fructose-2, 6-bisphosphatase gene (PFKFB3). Cytogenetic and Genome Research.

